# A rapid and systematic review of the effectiveness of temozolomide for the treatment of recurrent malignant glioma

**DOI:** 10.1038/sj.bjc.6600135

**Published:** 2002-02-12

**Authors:** J Dinnes, C Cave, S Huang, R Milne

**Affiliations:** Southampton Health Technology Assessment Centre, Wessex Institute for Health Research and Development, University of Southampton, Mailpoint 728, Boldrewood, Southampton SO16 7PX, UK

**Keywords:** temozolomide, chemotherapy, high-grade glioma, glioblastoma multiforme, anaplastic astrocytoma, systematic review

## Abstract

A rapid and systematic review of the effectiveness and cost-effectiveness of temozolomide in the treatment of recurrent malignant glioma was commissioned by the NHS HTA Programme on behalf of NICE. The full report has been published elsewhere. This paper summarizes the results for the effectiveness of temozolomide in people with recurrent glioblastoma multiforme and anaplastic astrocytoma. The review was conducted using standard systematic review methodology involving a systematic literature search, quality assessment of included studies with systematic data extraction and data synthesis. One randomized controlled trial and four uncontrolled studies were identified for inclusion. The key results were that temozolomide may increase progression-free survival but has no significant impact on overall length of survival. The main effect from temozolomide may have been in those patients who had not received any prior chemotherapy regimens, however further randomized controlled trials are required to confirm this suggestion. Temozolomide appears to produce few serious adverse effects and may also have a positive impact on health-related quality of life. Overall the evidence-base is weak and few strong conclusions can be drawn regarding the effectiveness of temozolomide. Large, well-designed randomized controlled trails conducted in a wider patient population are needed.

*British Journal of Cancer* (2002) **86**, 501–505. DOI: 10.1038/sj/bjc/6600135 www.bjcancer.com

© 2002 Cancer Research UK

## 

Temozolomide (Temodal®) was licensed in early 1999 for the treatment of patients with malignant glioma showing recurrence or progression after standard therapy (The European Agency for the Evaluation of Medicinal Products, 1999). Current treatment options for people with recurrent high-grade glioma (such as anaplastic astrocytoma (AA) or glioblastoma multiforme (GBM)) are limited, and life expectancy is short (Wong *et al*, 1999). In the UK, approximately one-third of patients currently receive chemotherapy, usually consisting of a single agent nitrosourea (e.g., CCNU or BCNU) or a combination therapy such as PCV (procarbazine, CCNU and vincristine) (Rampling, 2000). The success of these agents is thought to be limited, leading to considerable interest in newer chemotherapy agents such as temozolomide.

Temozolomide is claimed to be more effective, easier to administer and have fewer side-effects than its competitors. However, a lack of consensus regarding its true effectiveness and the relative expense of the drug has led to variations in its use across the country.

In mid-2000 the National Institute for Clinical Excellence (NICE) was asked to advise the NHS on ‘the use of temozolomide in brain cancer’. Our unit was commissioned by the NHS Health Technology Assessment Programme to conduct a systematic review to help inform the NICE decision. This paper presents the systematic review of the effectiveness literature only. The full report (available in the HTA monograph series) also presents the economic evaluation that was conducted (Dinnes *et al*, 2001).

The objective of this systematic review was therefore, to evaluate temozolomide for its licensed indications, in comparison to standard alternative chemotherapy or against best standard care, in terms of both survival and quality of life. No existing or ongoing systematic reviews of temozolomide were identified on the Cochrane Library prior to undertaking the review.

## MATERIALS AND METHODS

Studies were included if they assessed the effectiveness of temozolomide in brain cancer and were either a randomized controlled trial (RCT) or, if non-randomized, included over 45 patients. An extensive literature search was conducted using the generic and trade names for temozolomide in electronic databases (Cochrane Library, Medline, Embase, Cancerlit and Toxline) and by scanning reference lists of all retrieved papers. The authors of included studies were contacted to supply additional or missing data.

Study quality was assessed using a shortened version (Dinnes *et al*, 2001) of a checklist developed originally for an epidemiological review (Spitzer *et al*, 1990). The scale developed by Jadad *et al* (1996) was used to assess RCTs.

Two reviewers independently assessed studies for inclusion, extracted data and evaluated the quality of each included study. Disagreements were resolved through discussion.

Because of the paucity of data, only a narrative synthesis was undertaken. The results are summarized according to type of malignant glioma and outcome measures assessed. For ease of comparison all survival times initially reported in months are reported here in weeks (using the formula: weeks= (months×30.4)/7). All results have been rounded to one decimal point.

## RESULTS

Eight full reports of six studies were identified for inclusion in the main review (Dinnes *et al*, 2001). Only the five studies providing effectiveness data for temozolomide in patients with AA or GBM are reported here ( Table 1

**Table 1:**
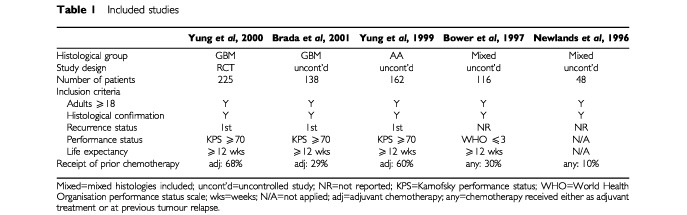
Included studies

). A full list of excluded studies is available on request from the authors. Figure 1

**Figure 1:**
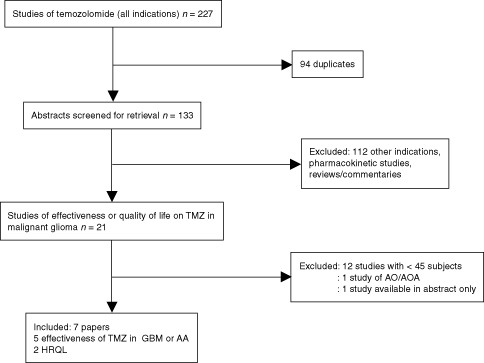
Flow diagram of temozolomide effectiveness search results.

provides an overview of the primary search and inclusion process.

### Description of included effectiveness studies

Only one RCT was identified (Yung *et al*, 2000). The remaining studies were uncontrolled (Newlands *et al*, 1996; Bower *et al*, 1997; Yung *et al*, 1999; Brada *et al*, 2001).

All studies applied similar inclusion criteria (Table 1). All included both patients who had received prior chemotherapy (as adjuvant treatment or at first recurrence) and patients who were chemotherapy-naïve.

Dosage of temozolomide was the same in all studies (200 mg m^−2^ day^−1^ for 5 days in each 28-day cycle). In patients who had received prior chemotherapy, the initial dose was 150 mg m^−2^ day^−1^, escalating to 200 mg m^−2^ day^−1^ after the first cycle if haematology results were satisfactory. The RCT compared temozolomide to procarbazine administered at a dosage of 150 mg m^−2^ day^−1^ for 28 consecutive days in each 56-day cycle.

### Quality of included studies

The method of randomization used in the RCT was not reported, leaving the potential for inadequate concealment of allocation of participants to treatment groups, but there did not appear to be substantial differences in baseline characteristics between the groups (Yung *et al*, 2000). Temozolomide patients on average had a shorter time from diagnosis to recurrence, but this was not found to have affected the results. It might reasonably be assumed that any bias introduced by a shorter time to recurrence would lead to poorer outcomes in the temozolomide group rather than exaggerating any potential benefit.

None of the studies give any assurance that clinicians and patients were blinded to the treatments given (and indeed this would not be possible in an uncontrolled study). This knowledge is likely to have affected assessments of clinical status and patients' self-reports of quality of life.

### Outcome measures

Several outcome measures are commonly reported in cancer trials including objective response, progression-free survival (PFS) and overall survival. Tumour response is of uncertain usefulness as an outcome measure in gliomas (Galanis and Buckner, 2000) and is not reported here. More important is the assessment of survival (progression free or overall). Six-month PFS was the primary outcome in most of the effectiveness studies. Median survival times have also been reported.

Several studies estimated survival using the Kaplan–Meier method, which allows estimation when there are censored observations. It should be noted, however, that survival proportions derived from Kaplan–Meier curves are estimated survival proportions at one single time point (e.g. 6 months), as opposed to a comparison of the total survival experience of the two groups. Hazard ratios are a more reliable means of comparing survival between groups.

Given the poor prognosis for malignant glioma, effects on health related quality of life (HRQL) should be considered. The EORTC QLQ-C30 instrument was used to assess quality of life in these studies.

More detailed discussion of factors that affect various outcome measures and how the included studies addressed these factors can be found in the full report (Dinnes *et al*, 2001).

### Assessment of effectiveness – Glioblastoma Multiforme

#### Progression-free survival

Results from the RCT (Yung *et al*, 2000) indicate that PFS was better in the temozolomide group, in terms of both the proportion of patients progression-free at 6 months (13% higher) and median PFS (4 weeks longer) (see Table 2

**Table 2:**
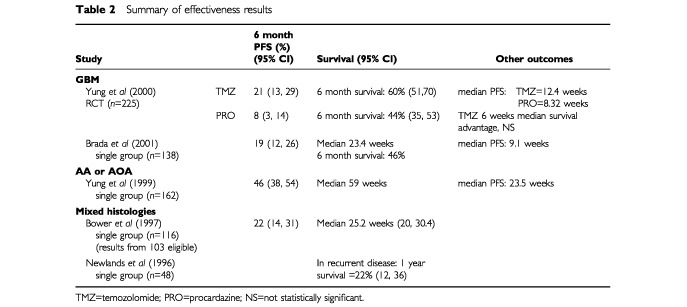
Summary of effectiveness results

). The statistical significance of these results was not provided. The number needed to treat (NNT) to achieve an extra progression-free patient at 6 months was 8 (95% CI: 5, 23 calculated by the authors).

The hazard ratio for the whole data set for PFS was 1.54 (CIs not provided), and for median PFS was 1.47 (95% CI: 1.11, 1.95).

In the uncontrolled study, 6-month PFS was 19% (95% CI: 12, 26%) and median PFS was 9.1 weeks for the whole sample and 9.6 weeks for those who were chemotherapy-naïve (*n*=98) (Brada *et al*, 2001).

#### Survival

The increases in overall survival demonstrated in the RCT were not found to be significant, although the logrank test indicated that there may have been meaningful differences across the groups (*P*=0.019). The reported nonsignificant difference in median survival was 1.5 months (Yung *et al*, 2000). Median survival in the uncontrolled study was 23.4 weeks (Brada *et al*, 2001).

#### Health-related quality of life

Patients receiving temozolomide in the RCT who remained progression free at 6 months showed improvements in five of seven pre-selected quality of life domains, although only one effect size was greater than 0.5 and only one was statistically significant (Osoba *et al*, 2000a). Patients receiving temozolomide in the uncontrolled study who remained progression free at 6 months showed improvements in all seven pre-selected domains. In this case, all seven effect sizes were less than 0.5 (usually considered small) and only three were statistically significant (Osoba *et al*, 2000a). In contrast, the patients receiving procarbazine in the RCT who remained progression-free, reported diminished HRQL in six domains, though none were statistically significant.

Progression of disease tended to lead to deterioration in HRQL scores across all groups, regardless of treatment. However, in temozolomide groups, there were improvements from baseline in the weeks preceding progression.

### Anaplastic astrocytoma

#### Survival

One uncontrolled study was found with results largely for people with AA (Yung *et al*, 1999). Six month PFS was better than for those with GBM at 46%, with a median length of survival of 23.5 weeks (Table 2). Results for those who were chemotherapy-naïve were not significantly better. Median survival from recurrence was 59 weeks for the whole sample and 50 weeks for the chemotherapy-naïve group.

#### Health-related quality of life

HRQL results in AA were similar to those for GBM. Those patients who remained progression free at 6 months showed improvements in all seven pre-selected quality of life domains (Osoba *et al*, 2000b). However, effect sizes were again small (less than 0.5) and reached statistical significance in only two domains.

### Mixed histologies

Limited results were available from the two studies conducted in mixed histological groups. One study reported 6-month PFS of 22% (95% CI: 14, 31%) (Newlands *et al*, 1996). The other reported a median survival of 25.2 weeks (Bower *et al*, 1997).

### Adverse effects of temozolomide

Myelosuppression is the most serious adverse effect of temozolomide and is dose limiting; however, it does not appear to be cumulative and is relatively easily treated. Between 6 and 10% of patients suffered grade 3 or 4 thrombocytopenia, less than 5% suffered each of grade 3 or 4 neutropenia, leukopenia or anaemia.

A wide range of other grade 3 or 4 adverse effects were noted in one study (Yung *et al*, 1999), most commonly asthenia (6%), headache (6%), nausea (10%) and vomiting (6%). These occurred in fewer patients in other studies. All studies routinely included anti-emetics or allowed their use as needed, noting that vomiting was generally well controlled by them.

In the RCT, the myelosuppressive effects were similar for both drugs, but nausea, vomiting and fatigue were noted more often in the procarbazine group (Yung *et al*, 2000). The number of adverse effects is affected by the number of cycles administered and length of treatment: over 90% of patients on temozolomide were treated for more than one cycle compared to only 33% of procarbazine patients.

## DISCUSSION

### Main results

Evidence to date, though limited, indicates that recurrent malignant glioma has some response to temozolomide. This response appears to be greater in AA than in GBM.

The main benefit in patients with GBM, demonstrated in one RCT and one relatively large uncontrolled study, is an increase (13%) in the estimated proportion of patients remaining progression-free at 6 months and a significant increase in median PFS of approximately 4 weeks. However, there was no significant overall survival advantage.

For patients with AA, one large uncontrolled study suggests favourable PFS and possibly increased overall survival. The magnitude of any benefit in AA is difficult to quantify due to the lack of a within study comparison of temozolomide with an alternative treatment regimen.

Temozolomide appears to involve few serious adverse effects. Vomiting appears to be well controlled by prophylactic anti-emetic regimens. Some clinicians believe that toxicity, particularly myelosuppression, is more predictable with temozolomide and this has been noted as one of the advantages of this drug over others. It should be noted, however, that little empirical evidence is available to support this position.

Temozolomide may appear to improve HRQL from recurrence until at or near disease progression and may confer considerably better quality of life than procarbazine. Given the cognitive impairments associated with brain tumours these improvements may be important in the daily functioning of patients and in their relationships with family and friends.

The incidence of AA and GBM in the UK is around three to four per 100 000 (Office for National Statistics, 2000). Current direct costs of treating malignant glioma in the UK are about £25 million per annum. Approximately 30% of patients have been considered for chemotherapy in the past. If this proportion were to be maintained, then around 600 patients per year could be eligible to receive temozolomide at an annual incremental cost to the NHS of about £4 million (Dinnes *et al*, 2001).

### Assumptions, limitations and uncertainties

This review was conducted rigorously and provides a balanced assessment of the current evidence. The search was extensive and it is very unlikely that RCTs were missed. It is also likely that all relevant uncontrolled studies were included, but the possibility here of publication bias is slightly greater. The conclusions that can be drawn from this review are however tentative and should be treated with caution, because of limitations in the evidence.

There were problems with the validity of the studies. Uncontrolled studies were eligible for inclusion in the review because of the anticipated lack of evidence from RCTs. Such studies, however, can only provide a broad indication of the potential effect of a treatment; they do not permit valid comparisons with alternative treatments. The only RCT found had several problems. It was conducted only in patients with GBM, did not use a comparator that is common in the UK, and was not powered to detect a clinically significant difference in outcomes. Limited details of the methods used in the trial, including methods of randomization, were available.

The reporting of results was problematic. First, increases in survival were presented as median rather than mean figures. Median results may provide a distorted summary of average increases in survival times when a treatment increases the life expectancy of some patients by a few weeks but has little or no impact on the survival of the patients who would otherwise live longest. Second, although in most studies radiological data were centrally reviewed and often by masked reviewers, there remained scope for biased assessment of outcomes, as many of the outcome measures used – particularly those evaluating tumour response – were subjective. None of the studies reviewed (including the RCT) used single or double-binding, largely due to the uncontrolled nature of the studies. Subjective clinical assessments and patients' self-reported quality of life may have been affected by knowledge of the treatment.

The generalizability of the results from the included studies is questionable, for three reasons. First, procarbazine was chosen as the comparator in the RCT because it is administered orally and is one of the few options available to patients who have recurrent glioma, particularly if they have had previous nitrosourea therapy. However, it is not commonly used alone in the UK, but instead is often used in combination therapy (PCV), at a lower dose than in the trial. Second, the method of recruiting subjects was not reported in three of the four uncontrolled studies (Bower *et al*, 1997; Yung *et al*, 1999; Brada *et al*, 2001). This may have led to the patients enrolled not being representative of the wider population of patients. Moreover, the performance status and life expectancy criteria applied in the studies will have led to the selection of patients who may have been somewhat healthier than those who would be offered temozolomide in practice. Third, the effect of temozolomide in patients who have undergone prior chemotherapy regimens compared to those who are chemotherapy naïve has yet to be established. It is plausible that prior chemotherapy should affect patients' response to future chemotherapy regimens, but evidence to date is based only on analyses in both subgroups of patients.

### Need for further research

Considerable research on temozolomide is ongoing, consisting mainly of uncontrolled studies in relatively small patient groups. The most pressing need is for adequately powered multi-centre RCTs that compare temozolomide for recurrent glioma with best alternative care such as PCV. These should recruit a large proportion of a wider population of patients (i.e. not limited to those with best prognosis) and be stratified by receipt of prior chemotherapy. Some of these research needs may be fulfilled by current ongoing or planned trials, although these are not expected to complete recruitment for several years (Dinnes *et al*, 2001).

## CONCLUSIONS

In summary, there is some indication of benefit from temozolomide, but the evidence is too weak for firm conclusions to be drawn and further large RCTs are required. The incidence of malignant glioma is relatively low and the overall budgetary impact for the NHS as a whole is in the order of £4 million per annum.

## ADDENDUM

Since this article was submitted for publication, a further uncontrolled study has been published that would meet the inclusion criteria for our review. This study evaluated temozolomide in 56 patients with recurrent glioblastoma multiforme, anaplastic astrocytoma or anaplastic oligodendrogliomas (Harris *et al*, 2001). The results were very similar to those in the other studies identified and would not change the overall conclusions of our review. Median progression free survival was 26 weeks and median survival was 48 weeks; results were substantially worse for those with glioblastoma multiforme in comparison to the other histologies.

## References

[bib1] BowerMNewlandsESBleehenNMBradaMBegentRJCalvertHColquhounILewisPBramptonMH1997 Multicentre CRC phase II trial of temozolomide in recurrent or progressive high-grade glioma Cancer Chemother Pharmacol 40 484488 9332462 10.1007/s002800050691

[bib2] BradaMHoang-XuanKRamplingRDietrichP-YDirixLMacdonaldDHeimansJZonnenbergBBravo-MarquesJHenrikssonRStuppRYueNBrunerJDuganMRaoSZaknoenS2001 Multicenter phase II trial of temozolomide in patients with glioblastoma multiforme at first relapse Ann Oncol 12 259266 11300335 10.1023/a:1008382516636

[bib3] DinnesJCaveCBHuangSMajorKMilneRM2001 The effectiveness and cost-effectiveness of temozolomide for the treatment of recurrent malignant glioma Health Technol Assess 513173 10.3310/hta5130 11359682

[bib4] GalanisEBucknerJ2000 Chemotherapy for high-grade gliomas Br J Cancer 82 13711380 10780513 10.1054/bjoc.1999.1075 PMC2363368

[bib5] HarrisMTRosenthalMAAshleyDLCherL2001 An Australian experience with temozolomide for the treatment of recurrent high grade gliomas J Clin Neurosci 8 325327 11437571 10.1054/jocn.2000.0809

[bib6] JadadAMooreACarrollDJenkinsonCReynoldsDJGavaghanDMcQuayHJ1996 Assessing the quality of reports of randomized clinical trials: is blinding necessary? Control Clin Trials 17 112 8721797 10.1016/0197-2456(95)00134-4

[bib7] NewlandsESO'ReillySMGlaserMGBowerMEvansHBrockCBramptonMHColquhounILewisPRice-EdwardsJMIllingworthRDRichardsPG1996 The Charing Cross Hospital experience with temozolomide in patients with gliomas Eur J Cancer Part A 32 22362241 10.1016/s0959-8049(96)00258-4 9038604

[bib8] Office for National Statistics2000 Health Statistics Quarterly (Autumn)London: The Stationery Office National Statistics

[bib9] OsobaDBradaMYungWKPradosM2000a Health-related quality of life in patients treated with temozolomide versus procarbazine for recurrent glioblastoma multiforme J Clin Oncol 18 14811491 10735896 10.1200/JCO.2000.18.7.1481

[bib10] OsobaDBradaMYungWKAPradosM2000b Health-related quality of life in patients with anaplastic astrocytoma during treatment with temozolomide Eur J Cancer 36 17881795 10974627 10.1016/s0959-8049(00)00165-9

[bib11] RamplingR2000 CRC CancerStats: Brain CRC CancerStats

[bib12] SpitzerWOLawrenceVDalesRHillGArcherMCClarkPAbenheimLHardyJSampalisJPinfoldSPMorganPP1990 Links between passive smoking and disease; A best-evidence synthesis Clin Invest Med 13 1742 2138069

[bib13] The European Agency for the Evaluation of Medicinal Products1999 Committee for Proprietary Medicinal Products European Public Assessment Report (EPAR): Temodal

[bib14] WongETHessKRGleasonMJJaeckleKAKyritsisAPPradosMDLevinVAYungWKA1999 Outcomes and prognostic factors in recurrent glioma patients enrolled onto phase II clinical trials J Clin Oncol 17 25722578 10561324 10.1200/JCO.1999.17.8.2572

[bib15] YungWKAlbrightREOlsonJFredericksRFinkKPradosMDBradaMSpenceAHohlRJShapiroWGlantzMGreenbergHSelkerRGVickNARamplingRFriedmanHPhillipsPBrunerJYueNOsobaDZaknoenSLevinVA2000 A phase II study of temozolomide vs. procarbazine in patients with glioblastoma multiforme at first relapse Br J Cancer 83 588593 10944597 10.1054/bjoc.2000.1316 PMC2363506

[bib16] YungWKAPradosMDYayaTRRosenfeldSSBradaMFriedmanHSAlbrightROlsonJChangSMO'NeillAMFriedmanAHBrunerJYueNDuganMZaknoenSLevinVA1999 Multicenter phase II trial of temozolomide in patients with anaplastic astrocytoma or anaplastic oligoastrocytoma at first relapse J Clin Oncol 17 27622771 10561351 10.1200/JCO.1999.17.9.2762

